# 3-Methyl-2-(methyl­sulfan­yl)-5,5-diphenyl-3,5-di­hydro-4*H*-imidazol-4-one

**DOI:** 10.1107/S2414314623002080

**Published:** 2023-03-10

**Authors:** Abderrazzak El Moutaouakil Ala Allah, Walid Guerrab, Abdulsalam Alsubari, Joel T. Mague, Youssef Ramli

**Affiliations:** a Laboratory of Medicinal Chemistry, Drug Sciences Research Center, Faculty of Medicine and Pharmacy, Mohammed V University in Rabat, Morocco; b Laboratory of Medicinal Chemistry, Faculty of Clinical Pharmacy, 21 September University, Yemen; c Department of Chemistry, Tulane University, New Orleans, LA 70118, USA; Katholieke Universiteit Leuven, Belgium

**Keywords:** crystal structure, hydrogen bond, di­hydro­imidazolone, alkyl­ation

## Abstract

In the title mol­ecule, C_17_H_16_N_2_OS, the di­hydro­imidazolone ring is slightly puckered and the methyl­sulfanyl group is nearly coplanar with it. In the crystal, two sets of C—H⋯O hydrogen bonds form corrugated layers of mol­ecules parallel to the *ac* plane. The layers pack with normal van der Waals contacts between them.

## Abstract 2

In the title mol­ecule, C_17_H_16_N_2_OS, the di­hydro­imidazolone ring is slightly puckered and the methyl­sulfanyl group is nearly coplanar with it.

## Graphical abstract

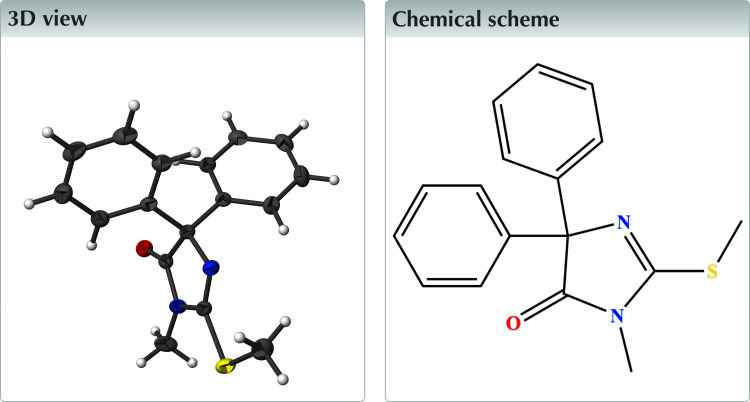

## Structure description

**Figure 1:**
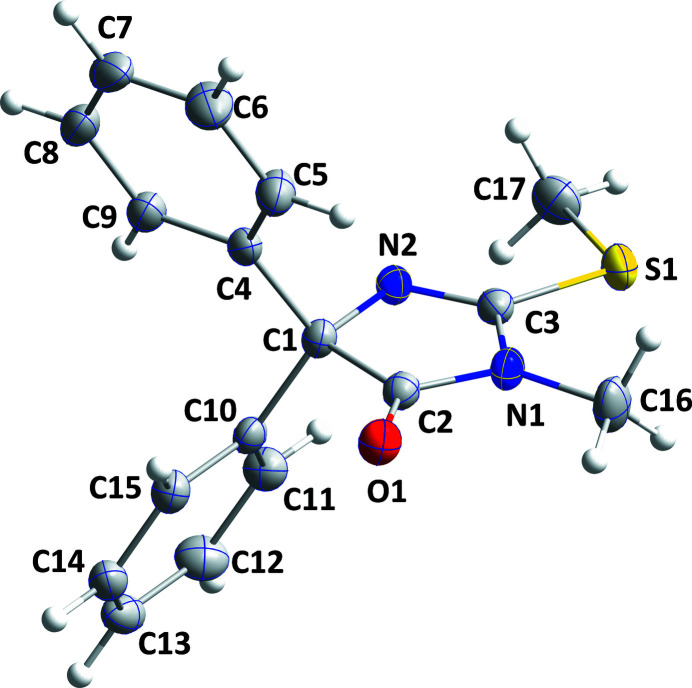
The title mol­ecule with the atom-labeling scheme and 50% probability ellipsoids.

Imidazole and its derivatives display various biological effects such as insecticides, herbicides and fungicides (Tutino *et al.*, 2009 ▸; Wu *et al.*, 2023 ▸; Takle *et al.*, 2006 ▸). Our team has been working on these derivatives for some years to evaluate their biological activities (*e.g.* Guerrab *et al.*, 2022*a* ▸,*b* ▸) and corrosion inhibition activities (*e.g.* Nabah et *al.*, 2020 ▸). In a continuation of our recent work focused on the synthesis and biological evaluation of phenytoin derivatives (*e.g.* Guerrab et *al.*, 2023 ▸), we report here the crystal structure of the title compound (Fig. 1 ▸).

**Figure 2:**
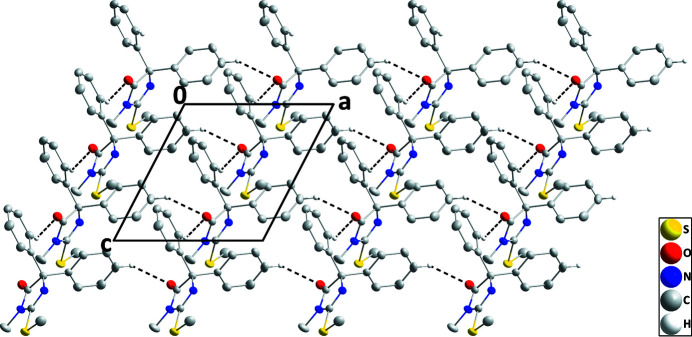
A portion of one layer viewed along the *b*-axis direction with C—H⋯O hydrogen bonds depicted by dashed lines. Non-inter­acting hydrogen atoms are omitted for clarity.

**Figure 3:**
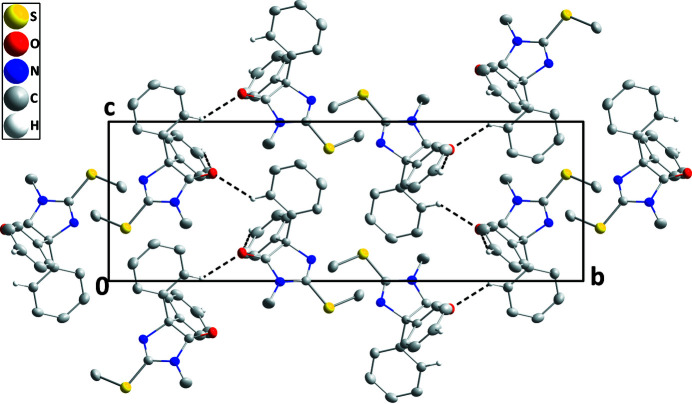
Packing viewed along the *a*-axis direction giving end views of parts of four layers with C—H⋯O hydrogen bonds depicted by dashed lines. Non-inter­acting hydrogen atoms are omitted for clarity.

The mol­ecule adopts the conformation typical for this class of mol­ecule with the phenyl groups projecting out above and below the plane of the di­hydro­imidazolone ring. The latter ring is slightly puckered [C1 and C2 deviate by −0.0122 (7) and 0.0121 (7) Å, respectively, from the mean plane] and the mean planes of the C4–C9 and C10–C15 rings are inclined to its mean plane by 72.32 (5) and 67.03 (3)°, respectively. The terminal carbon atom of the methyl­sulfanyl group lies close to the mean plane of the di­hydro­imidazolone ring, as indicated by the C17—S1—C3—N2 torsion angle of −2.75 (13)°. In the crystal, C7—H7⋯O1 hydrogen bonds (Table 1 ▸) form chains of mol­ecules extending along the *a*-axis direction. These are linked by C15—H15⋯O1 hydrogen bonds, forming corrugated layers of mol­ecules parallel to the *ac* plane (Table 1 ▸ and Fig. 2 ▸). The layers pack along the *b*-axis direction with normal van de Waals contacts (Fig. 3 ▸) between them.

## Synthesis and crystallization

Thio­hydantoin (1000 mg, 3.73 mmol) was placed in a flask with K_2_CO_3_ (1030 mg, 7.46 mmol) in 20 ml of absolute di­methyl­formamide (DMF), and two equivalents of iodo­methane (0.5 ml, 1160 mg) were added. The solution was left stirring for 2 h at room temperature. The reaction mixture was filtered, and the solvent was distilled off under reduced pressure. The residue obtained was recrystallized from methanol solution to yield colorless, plate-like single crystals (Akrad et *al*., 2018 ▸).

## Refinement

Crystal data, data collection and structure refinement details are summarized in Table 2 ▸.

## Supplementary Material

Crystal structure: contains datablock(s) global, I. DOI: 10.1107/S2414314623002080/vm4058sup1.cif

Structure factors: contains datablock(s) I. DOI: 10.1107/S2414314623002080/vm4058Isup2.hkl

Supporting information file. DOI: 10.1107/S2414314623002080/vm4058Isup3.cml

CCDC reference: 2246179

Additional supporting information:  crystallographic information; 3D view; checkCIF report

## Figures and Tables

**Table 1:** Hydrogen-bond geometry (Å, °)

*D*—H⋯*A*	*D*—H	H⋯*A*	*D*⋯*A*	*D*—H⋯*A*
C7—H7⋯O1^i^	0.95	2.55	3.390 (2)	148
C15—H15⋯O1^ii^	0.95	2.59	3.3797 (17)	140

Symmetry codes: (i) 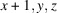 ; (ii) 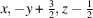 .

**Table 2:** Experimental details

Crystal data	
Chemical formula	C_17_H_16_N_2_OS
*M*_r_	296.38
Crystal system, space group	Monoclinic, *P*2_1_/*c*
Temperature (K)	150
*a*, *b*, *c* (Å)	8.4129 (1), 22.9217 (4), 8.6719 (1)
β (°)	117.417 (1)
*V* (Å^3^)	1484.44 (4)
*Z*	4
Radiation type	Cu *K*α
μ (mm^−1^)	1.93
Crystal size (mm)	0.20 × 0.06 × 0.03

Data collection	
Diffractometer	Bruker D8 VENTURE PHOTON 3 CPAD
Absorption correction	Multi-scan (*SADABS*; Krause *et al.*, 2015 ▸)
*T*_min_, *T*_max_	0.85, 0.94
No. of measured, independent and observed [*I* > 2σ(*I*)] reflections	37955, 2930, 2733
*R*_int_	0.036
(sin θ/λ)_max_ (Å^−1^)	0.618

Refinement	
*R*[*F* ^2^ > 2σ(*F* ^2^)], *wR*(*F* ^2^), *S*	0.030, 0.080, 1.04
No. of reflections	2930
No. of parameters	192
H-atom treatment	H-atom parameters constrained
Δρ_max_, Δρ_min_ (e Å^−3^)	0.30, −0.24

Computer programs: *APEX4* and *SAINT* (Bruker, 2021 ▸), *SHELXT* (Sheldrick, 2015*a* ▸), *SHELXL2019/1* (Sheldrick, 2015*b* ▸), *DIAMOND* (Brandenburg & Putz, 2012 ▸) and *SHELXTL* (Sheldrick, 2008 ▸).

## full crystallographic data

### Crystal data

**Table:**

C_17_H_16_N_2_OS	*F*(000) = 624
*M**_r_* = 296.38	*D*_x_ = 1.326 Mg m^−^^3^
Monoclinic, *P*2_1_/*c*	Cu *K*α radiation, λ = 1.54178 Å
*a* = 8.4129 (1) Å	Cell parameters from 9855 reflections
*b* = 22.9217 (4) Å	θ = 3.9–72.4°
*c* = 8.6719 (1) Å	µ = 1.93 mm^−^^1^
β = 117.417 (1)°	*T* = 150 K
*V* = 1484.44 (4) Å^3^	Plate, colourless
*Z* = 4	0.20 × 0.06 × 0.03 mm

### Data collection

**Table:**

Bruker D8 VENTURE PHOTON 3 CPAD diffractometer	2930 independent reflections
Radiation source: INCOATEC IµS micro—-focus source	2733 reflections with *I* > 2σ(*I*)
Mirror monochromator	*R*_int_ = 0.036
Detector resolution: 7.3910 pixels mm^-1^	θ_max_ = 72.4°, θ_min_ = 3.9°
φ and ω scans	*h* = −9→10
Absorption correction: multi-scan (*SADABS*; Krause *et al.*, 2015)	*k* = −28→28
*T*_min_ = 0.85, *T*_max_ = 0.94	*l* = −10→10
37955 measured reflections	

### Refinement

**Table:**

Refinement on *F*^2^	Primary atom site location: dual
Least-squares matrix: full	Secondary atom site location: difference Fourier map
*R*[*F*^2^ > 2σ(*F*^2^)] = 0.030	Hydrogen site location: inferred from neighbouring sites
*wR*(*F*^2^) = 0.080	H-atom parameters constrained
*S* = 1.04	*w* = 1/[σ^2^(*F*_o_^2^) + (0.040*P*)^2^ + 0.6009*P*] where *P* = (*F*_o_^2^ + 2*F*_c_^2^)/3
2930 reflections	(Δ/σ)_max_ = 0.001
192 parameters	Δρ_max_ = 0.30 e Å^−^^3^
0 restraints	Δρ_min_ = −0.24 e Å^−^^3^

### Special details

**Table:**

Experimental. The diffraction data were obtained from 15 sets of frames, each of width 0.5° in ω or φ, collected with scan parameters determined by the "strategy" routine in *APEX4*. The scan time was θ-dependent and ranged from 5 to 15 sec/frame.
Geometry. All esds (except the esd in the dihedral angle between two l.s. planes) are estimated using the full covariance matrix. The cell esds are taken into account individually in the estimation of esds in distances, angles and torsion angles; correlations between esds in cell parameters are only used when they are defined by crystal symmetry. An approximate (isotropic) treatment of cell esds is used for estimating esds involving l.s. planes.
Refinement. Refinement of F^2^ against ALL reflections. The weighted R-factor wR and goodness of fit S are based on F^2^, conventional R-factors R are based on F, with F set to zero for negative F^2^. The threshold expression of F^2^ > 2sigma(F^2^) is used only for calculating R-factors(gt) etc. and is not relevant to the choice of reflections for refinement. R-factors based on F^2^ are statistically about twice as large as those based on F, and R- factors based on ALL data will be even larger. H-atoms attached to carbon were placed in calculated positions (C—H = 0.95 - 0.98 Å). All were included as riding contributions with isotropic displacement parameters 1.2 - 1.5 times those of the attached atoms.

### Fractional atomic coordinates and isotropic or equivalent isotropic displacement parameters (Å^2^)

**Table:**

	*x*	*y*	*z*	*U*_iso_*/*U*_eq_	
S1	0.72407 (4)	0.53586 (2)	1.16914 (4)	0.02823 (11)	
O1	0.54051 (12)	0.71912 (4)	0.82460 (12)	0.0271 (2)	
N1	0.61671 (14)	0.63912 (5)	1.00499 (13)	0.0236 (2)	
N2	0.70761 (14)	0.57313 (4)	0.86599 (13)	0.0217 (2)	
C1	0.64968 (16)	0.62639 (5)	0.75658 (15)	0.0202 (2)	
C2	0.59464 (16)	0.66941 (5)	0.86029 (16)	0.0216 (2)	
C3	0.68352 (15)	0.58379 (5)	0.99846 (15)	0.0216 (2)	
C4	0.80769 (16)	0.64949 (5)	0.73319 (16)	0.0219 (3)	
C5	0.92075 (18)	0.69236 (6)	0.84133 (18)	0.0305 (3)	
H5	0.896928	0.709387	0.928442	0.037*	
C6	1.0688 (2)	0.71050 (7)	0.8226 (2)	0.0369 (3)	
H6	1.145882	0.739664	0.897666	0.044*	
C7	1.10450 (18)	0.68648 (7)	0.69606 (19)	0.0346 (3)	
H7	1.205289	0.699222	0.683353	0.041*	
C8	0.99264 (19)	0.64370 (6)	0.58764 (18)	0.0321 (3)	
H8	1.016772	0.626991	0.500337	0.039*	
C9	0.84503 (17)	0.62513 (6)	0.60624 (17)	0.0260 (3)	
H9	0.769091	0.595615	0.531833	0.031*	
C10	0.48418 (16)	0.61446 (5)	0.58351 (15)	0.0209 (2)	
C11	0.39872 (18)	0.56066 (6)	0.54725 (17)	0.0273 (3)	
H11	0.443592	0.530142	0.630553	0.033*	
C12	0.24745 (19)	0.55132 (6)	0.38914 (19)	0.0337 (3)	
H12	0.190002	0.514342	0.364685	0.040*	
C13	0.18031 (17)	0.59563 (6)	0.26740 (17)	0.0301 (3)	
H13	0.076931	0.589203	0.159682	0.036*	
C14	0.26501 (17)	0.64947 (6)	0.30376 (17)	0.0277 (3)	
H14	0.218962	0.680058	0.220802	0.033*	
C15	0.41649 (17)	0.65893 (6)	0.46046 (16)	0.0249 (3)	
H15	0.474305	0.695842	0.484019	0.030*	
C16	0.5778 (2)	0.66282 (7)	1.13960 (19)	0.0364 (3)	
H16A	0.690265	0.671538	1.243296	0.055*	
H16B	0.509363	0.634189	1.168803	0.055*	
H16C	0.507500	0.698691	1.097364	0.055*	
C17	0.7916 (2)	0.47276 (6)	1.0905 (2)	0.0348 (3)	
H17A	0.700908	0.464056	0.971597	0.052*	
H17B	0.804208	0.439295	1.165589	0.052*	
H17C	0.906603	0.480549	1.091373	0.052*	

### Atomic displacement parameters (Å^2^)

**Table:**

	*U*^11^	*U*^22^	*U*^33^	*U*^12^	*U*^13^	*U*^23^
S1	0.03234 (19)	0.03103 (18)	0.02251 (17)	0.00075 (12)	0.01364 (14)	0.00651 (12)
O1	0.0333 (5)	0.0212 (4)	0.0296 (5)	0.0024 (4)	0.0169 (4)	−0.0003 (4)
N1	0.0283 (5)	0.0254 (5)	0.0210 (5)	0.0013 (4)	0.0146 (4)	−0.0003 (4)
N2	0.0236 (5)	0.0217 (5)	0.0209 (5)	0.0003 (4)	0.0112 (4)	0.0020 (4)
C1	0.0232 (6)	0.0197 (5)	0.0202 (6)	0.0001 (4)	0.0120 (5)	0.0006 (4)
C2	0.0207 (6)	0.0239 (6)	0.0210 (6)	−0.0026 (5)	0.0102 (5)	−0.0018 (5)
C3	0.0201 (6)	0.0240 (6)	0.0206 (6)	−0.0016 (4)	0.0092 (5)	0.0011 (5)
C4	0.0213 (6)	0.0249 (6)	0.0199 (6)	0.0016 (4)	0.0098 (5)	0.0046 (5)
C5	0.0308 (7)	0.0374 (7)	0.0256 (6)	−0.0069 (6)	0.0150 (6)	−0.0020 (5)
C6	0.0305 (7)	0.0444 (8)	0.0340 (8)	−0.0126 (6)	0.0132 (6)	0.0001 (6)
C7	0.0235 (6)	0.0466 (8)	0.0359 (8)	0.0009 (6)	0.0157 (6)	0.0139 (6)
C8	0.0300 (7)	0.0418 (8)	0.0312 (7)	0.0081 (6)	0.0199 (6)	0.0080 (6)
C9	0.0262 (6)	0.0292 (6)	0.0250 (6)	0.0030 (5)	0.0137 (5)	0.0022 (5)
C10	0.0216 (6)	0.0239 (6)	0.0208 (6)	0.0007 (4)	0.0130 (5)	−0.0014 (4)
C11	0.0279 (6)	0.0237 (6)	0.0297 (7)	−0.0007 (5)	0.0129 (5)	0.0006 (5)
C12	0.0295 (7)	0.0294 (7)	0.0371 (8)	−0.0057 (5)	0.0111 (6)	−0.0065 (6)
C13	0.0231 (6)	0.0398 (7)	0.0250 (6)	0.0013 (5)	0.0090 (5)	−0.0068 (5)
C14	0.0274 (7)	0.0334 (7)	0.0229 (6)	0.0056 (5)	0.0122 (5)	0.0030 (5)
C15	0.0280 (6)	0.0246 (6)	0.0245 (6)	−0.0005 (5)	0.0141 (5)	0.0003 (5)
C16	0.0501 (9)	0.0395 (8)	0.0285 (7)	0.0082 (6)	0.0257 (7)	−0.0008 (6)
C17	0.0381 (8)	0.0278 (7)	0.0356 (8)	0.0051 (6)	0.0145 (6)	0.0074 (6)

### Geometric parameters (Å, º)

**Table:**

S1—C3	1.7461 (12)	C8—H8	0.9500
S1—C17	1.7995 (15)	C9—H9	0.9500
O1—C2	1.2130 (15)	C10—C11	1.3887 (17)
N1—C2	1.3699 (16)	C10—C15	1.3937 (17)
N1—C3	1.3991 (16)	C11—C12	1.3922 (19)
N1—C16	1.4550 (16)	C11—H11	0.9500
N2—C3	1.2786 (16)	C12—C13	1.384 (2)
N2—C1	1.4840 (15)	C12—H12	0.9500
C1—C4	1.5287 (16)	C13—C14	1.387 (2)
C1—C10	1.5302 (16)	C13—H13	0.9500
C1—C2	1.5426 (16)	C14—C15	1.3867 (19)
C4—C5	1.3888 (18)	C14—H14	0.9500
C4—C9	1.3932 (18)	C15—H15	0.9500
C5—C6	1.391 (2)	C16—H16A	0.9800
C5—H5	0.9500	C16—H16B	0.9800
C6—C7	1.379 (2)	C16—H16C	0.9800
C6—H6	0.9500	C17—H17A	0.9800
C7—C8	1.384 (2)	C17—H17B	0.9800
C7—H7	0.9500	C17—H17C	0.9800
C8—C9	1.3907 (19)		
			
C3—S1—C17	99.02 (6)	C8—C9—H9	119.8
C2—N1—C3	108.05 (10)	C4—C9—H9	119.8
C2—N1—C16	124.06 (11)	C11—C10—C15	119.33 (12)
C3—N1—C16	127.88 (11)	C11—C10—C1	121.54 (11)
C3—N2—C1	106.02 (10)	C15—C10—C1	119.12 (11)
N2—C1—C4	108.67 (9)	C10—C11—C12	120.18 (12)
N2—C1—C10	111.27 (9)	C10—C11—H11	119.9
C4—C1—C10	112.70 (10)	C12—C11—H11	119.9
N2—C1—C2	104.57 (9)	C13—C12—C11	120.34 (13)
C4—C1—C2	111.64 (10)	C13—C12—H12	119.8
C10—C1—C2	107.71 (9)	C11—C12—H12	119.8
O1—C2—N1	125.90 (11)	C12—C13—C14	119.54 (12)
O1—C2—C1	129.05 (11)	C12—C13—H13	120.2
N1—C2—C1	105.04 (10)	C14—C13—H13	120.2
N2—C3—N1	116.26 (11)	C15—C14—C13	120.41 (12)
N2—C3—S1	126.26 (10)	C15—C14—H14	119.8
N1—C3—S1	117.47 (9)	C13—C14—H14	119.8
C5—C4—C9	118.93 (12)	C14—C15—C10	120.20 (12)
C5—C4—C1	121.51 (11)	C14—C15—H15	119.9
C9—C4—C1	119.49 (11)	C10—C15—H15	119.9
C4—C5—C6	120.28 (13)	N1—C16—H16A	109.5
C4—C5—H5	119.9	N1—C16—H16B	109.5
C6—C5—H5	119.9	H16A—C16—H16B	109.5
C7—C6—C5	120.55 (14)	N1—C16—H16C	109.5
C7—C6—H6	119.7	H16A—C16—H16C	109.5
C5—C6—H6	119.7	H16B—C16—H16C	109.5
C6—C7—C8	119.62 (13)	S1—C17—H17A	109.5
C6—C7—H7	120.2	S1—C17—H17B	109.5
C8—C7—H7	120.2	H17A—C17—H17B	109.5
C7—C8—C9	120.14 (13)	S1—C17—H17C	109.5
C7—C8—H8	119.9	H17A—C17—H17C	109.5
C9—C8—H8	119.9	H17B—C17—H17C	109.5
C8—C9—C4	120.49 (13)		
			
C3—N2—C1—C4	121.22 (11)	C10—C1—C4—C9	−41.41 (15)
C3—N2—C1—C10	−114.13 (11)	C2—C1—C4—C9	−162.79 (11)
C3—N2—C1—C2	1.88 (12)	C9—C4—C5—C6	0.0 (2)
C3—N1—C2—O1	−179.29 (12)	C1—C4—C5—C6	176.77 (12)
C16—N1—C2—O1	−0.1 (2)	C4—C5—C6—C7	0.4 (2)
C3—N1—C2—C1	1.68 (12)	C5—C6—C7—C8	−0.4 (2)
C16—N1—C2—C1	−179.13 (12)	C6—C7—C8—C9	0.1 (2)
N2—C1—C2—O1	178.84 (12)	C7—C8—C9—C4	0.3 (2)
C4—C1—C2—O1	61.52 (16)	C5—C4—C9—C8	−0.33 (19)
C10—C1—C2—O1	−62.70 (16)	C1—C4—C9—C8	−177.20 (12)
N2—C1—C2—N1	−2.18 (12)	N2—C1—C10—C11	4.07 (15)
C4—C1—C2—N1	−119.50 (11)	C4—C1—C10—C11	126.43 (12)
C10—C1—C2—N1	116.28 (10)	C2—C1—C10—C11	−109.99 (12)
C1—N2—C3—N1	−0.95 (14)	N2—C1—C10—C15	−176.77 (10)
C1—N2—C3—S1	178.44 (9)	C4—C1—C10—C15	−54.41 (14)
C2—N1—C3—N2	−0.54 (15)	C2—C1—C10—C15	69.17 (13)
C16—N1—C3—N2	−179.68 (13)	C15—C10—C11—C12	0.28 (19)
C2—N1—C3—S1	−179.98 (8)	C1—C10—C11—C12	179.44 (12)
C16—N1—C3—S1	0.87 (18)	C10—C11—C12—C13	−0.4 (2)
C17—S1—C3—N2	−2.75 (13)	C11—C12—C13—C14	0.1 (2)
C17—S1—C3—N1	176.63 (10)	C12—C13—C14—C15	0.3 (2)
N2—C1—C4—C5	−94.40 (14)	C13—C14—C15—C10	−0.50 (19)
C10—C1—C4—C5	141.79 (12)	C11—C10—C15—C14	0.19 (18)
C2—C1—C4—C5	20.42 (16)	C1—C10—C15—C14	−178.99 (11)
N2—C1—C4—C9	82.39 (13)		

### Hydrogen-bond geometry (Å, º)

**Table:**

*D*—H···*A*	*D*—H	H···*A*	*D*···*A*	*D*—H···*A*
C7—H7···O1^i^	0.95	2.55	3.390 (2)	148
C15—H15···O1^ii^	0.95	2.59	3.3797 (17)	140

Symmetry codes: (i) *x*+1, *y*, *z*; (ii) *x*, −*y*+3/2, *z*−1/2.
